# The Academic Manifesto: From an Occupied to a Public University

**DOI:** 10.1007/s11024-015-9270-9

**Published:** 2015-04-03

**Authors:** Willem Halffman, Hans Radder

**Affiliations:** 1 Institute for Science, Innovation and Society, Faculty of Science, Mailbox 77, Radboud University Nijmegen, P.O. Box 9010, 6500 GL Nijmegen, The Netherlands; 2 Department of Philosophy, VU University Amsterdam, Amsterdam, The Netherlands

**Keywords:** Academic politics, Management, The Netherlands, Public university, Academic labour conditions, Political action

## Abstract

Universities are occupied by management, a regime obsessed with ‘accountability’ through measurement, increased competition, efficiency, ‘excellence’, and misconceived economic salvation. Given the occupation’s absurd side-effects, we ask ourselves how management has succeeded in taking over our precious universities. An alternative vision for the academic future consists of a public university, more akin to a socially engaged knowledge commons than to a corporation. We suggest some provocative measures to bring about such a university. However, as management seems impervious to cogent arguments, such changes can only happen if academics take action. Hence, we explore several strategies for a renewed university politics.

## The Occupied University

The university has been occupied – not by students demanding a say (as in the 1960s), but this time by the many-headed Wolf of management.^1^ The Wolf has colonised academia with a mercenary army of professional administrators, armed with spreadsheets, output indicators and audit procedures, loudly accompanied by the Efficiency and Excellence March. Management has proclaimed academics the enemy within: academics cannot be trusted, and so have to be tested and monitored, under the permanent threat of reorganisation, termination and dismissal. The academics allow themselves to be meekly played off against one another, like frightened, obedient sheep, hoping to make it by staying just ahead of their colleagues. The Wolf uses the most absurd means to remain in control, such as money-wasting semi- and full mergers, increasingly detailed, and thus costly, accountability systems and extremely expensive prestige projects.

This conquest seems to work and the export of knowledge from the newly conquered colony can be ever increased, but inland the troubles fester. Thus, while all the glossed-up indicators constantly point to the stars, the mood on the academic shop floor steadily drops. The Wolf pops champagne after each new score in the Shanghai Competition, while the university sheep desperately work until they drop^2^ and the quality of the knowledge plantations is starting to falter, as is demonstrated by a large number of comprehensive and thorough analyses.^3^ Meanwhile, the sheep endeavour to bring the absurd anomalies of the occupation to the Wolf’s attention by means of an endless stream of opinion articles, lamentations, pressing letters and appeals. In turn, the Wolf reduces these to mere incidents, brushes them aside as inevitable side effects of progress, or simply ignores them.

The occupation may have a different shape and intensity in different places, but it cannot be reduced to a few isolated phenomena. It is rather a highly significant and pervasive pattern that applies in varying degrees to many universities in many countries. The fact that there may be other animals around does not make the presence of the Wolf less significant or threatening.

Although our description and evaluation are written from the perspective of Dutch universities, the gist of our account (and quite a few details) applies to other countries as well, especially in Europe.^4^ While management’s occupation may not be as advanced in the Netherlands as it is in England (Holmwood 2011), it has already established a powerful continental bridgehead (De Boer, Enders and Schimank 2007). To show how these developments are more than just incidents, we list six critical processes and their excesses below. We will then proceed to analyse causes and suggest remedies.^5^

### Measurability for Accountability

Under management’s occupation, scientists are measured against one another with endlessly changing yardsticks. The supposition is that this will make their complex work accountable to outsiders, make the ‘output’ fit the accountants’ spreadsheets. In the Netherlands, counting output started off with the number of publications, then international publications, after which only English-language publications counted, thereafter articles in high-impact journals, and eventually often-cited publications (leading to a high ‘h-index’).^6^ Because jobs and the survival of entire departments depend on these indicators, everyone does their best to buff up the scores, if need be at the expense of content. Academics assist their colleagues with citations to increase their h-index and travel endlessly to conferences to surpass one another in visibility with slick PR presentations. Journals demand references to their papers as a precondition for publication in order to increase their (senseless) impact factor (Weingart 2005; American Society for Cell Biology et al. 2012; Vanclay 2012; Dijstelbloem et al. 2013), while academics make cynical jokes about the ‘smallest publishable unit’. After the collapse of each indicator, we fabricate a new one and the game starts all over again.

The problem does not lie with the technical inadequacy of a particular indicator, but in the very regime of indicator fetishism itself. The regime does not really care about high-quality results, which it cannot judge, but rather about performance: the tactically well thought-out and cleverly buffed-up *illusion* of excellence. These indicators have fundamentally changed science itself. They ignore and destroy the variety of knowledge forms and practices in various fields of study. That what is not measurable and comparable, does not count, is a waste of energy and should therefore be destroyed. In the indicator game, a book of four hundred pages published by Cambridge University Press hardly counts, or does not even count at all; a three-page article does. The specific publication system of (a part of) the natural and life sciences has been forced upon the rest of the sciences, even where it does not fit.

The scientific publication system is now all but broken: it is caving in under an endless stream of worthless publications, edited papers posing as republications ‘for a different audience’, strategic citations, and opportunistic or commercial journals: an exponentially growing stream of output, hardly ever read. You do not further your career in this publication factory (Halffman and Leydesdorff 2010; Abma 2013) by *reading* all these papers, but rather by *writing* as many as possible, or at least by adding your name to them – and finding this absolutely normal.^7^

### Permanent Competition under the Pretext of ‘Quality’

Institutions are measured against other institutions, researchers compete with one another for funds and universities for students. This leads to a permanent state of war between all the parties, destroying the social fabric of the university, but benefiting the occupier. You can no longer present your research proposals to your colleagues in good faith, because they may appropriate your ideas. Your institute may not show any weakness at all, as this can result in its termination with the support of neighbouring departments. Of all tasks in the academic workplace, teaching is the least appreciated and has to be outsourced as soon as possible, allowing people to focus on the battle for coveted research money.

This competition is part of a culture of mistrust, which is explicitly applauded and encouraged: university employees are suspect, scroungers, parasites. The insinuation is that if they are not constantly threatened, they do nothing. The strong inner drive of many academics who, despite all these absurdities, stubbornly persevere is scornfully dismissed as unreliable.

Much is also expected from competition for students. Competition between universities and degree programmes, measured according to audits and rankings, is presumed to improve the quality of education. The student-consumer, however, does not opt for quality, but rather for an image, an attractive student life, or fancy facilities. Hence universities invest in appealing restaurants and sports facilities. And if the university or degree programme then grows (to the joy of the managers), the quality tends to decrease rather than increase, because the infrastructure of lecturing rooms, lecturers, teaching methods and administration cannot cope with the influx.^8^

This situation of permanent competition and mistrust subverts each impetus for organisation and collective resistance by lecturers and researchers opposing this system. Disposable staff are opportunistically left dangling with temporary contracts, often with an appeal to their sense of responsibility towards their students. Overtime work and structural job insecurity, including above-average stress levels, have become the rule (Gill 2009). Universities now tend to offer academic kamikaze contracts with just about 100% lecturing duties, often comprising far more work than is paid for. The carrot offered is the suggestion of more permanent academic employment in the future. In light of the fierce competition, this is only feasible for those who ‘qualify’ by obtaining research results in their unpaid free time. The defence of this system is that ‘Nobody is forced to sign such a contract’. A structural injustice is legitimised by making it an individual choice: ‘Exploitation? You are doing it to yourself’.

### The Promise of Greater ‘Efficiency’

In addition to quality improvement, the managerial university also claims to increase its efficiency. Hence we do not need to provide universities with more resources; we provide managers who will attract extra resources through increased efficiency, under the pressure of mutual competition. Competition will not only make the university better, but also cheaper – this is the promise.

However, in practice, competition between degree programmes is about slick communication and prestige projects – which are not cheap at all. One hilarious book (Tuchman 2009) describes the shift of resources from research to PR projects at an American university, but in the Netherlands things also go wrong. A typical faculty now has a solid communications team and also at the university level there are large divisions of marketing and communication. They produce high-priced newspaper advertisements containing the most recent, excellent external review results, regularly introduce a new corporate image, as well as sparkling websites. They plug Acclaimed Professors in TV talk shows in the hope of luring away students from the competitor. Providing real information about a degree programme is still voluntary work, by lecturers behind a table on a Saturday afternoon university information day or in a demonstration class at secondary schools.

Specifically in research, competition leads to outrageously high overheads. Writing research proposals requires a substantial part of the research time (Herbert, Barnett and Graves 2013), in an environment where the chances of a research application’s success are often one in ten, or even less. Researchers try their fortune with European funding agencies, even if it requires taking on its notorious bureaucracy, its Byzantine research programmes, its forced cooperation constructions, its lobbying in the Brussels corridors, and the expensive application-writing advisors at universities.

Similar processes occur in the Netherlands on a national scale. According to a conservative estimate, the overheads of writing, reviewing and allocating the NWO Veni subsidies (The ‘Innovational Research Incentives Scheme’ of the Netherlands Organisation for Scientific Research) amount to about a quarter of the research budget (Van Arensbergen, Hessels and Van der Meulen 2013: 47). Here too, there are costly preparation procedures with specialised advisors coaching applicants for the procedure. In the end, this expensive assessment system does not even succeed in selecting the best researchers (Van den Besselaar and Leydesdorff 2009). In spite of this, receiving such prestigious ‘talent subsidies’ is increasingly used as a criterion for further appointments at universities.

A considerable part of these overheads is shamelessly passed on to the primary process. Participating in this assessment circus is often a matter of working in the evening or during the weekend. This work is rarely paid and seldom interesting, apart from keeping track of what the competitors are up to. In practice, the real meaning of ‘efficiency’ and ‘quality’ is ‘working over the weekend again’.

Meanwhile, the overheads of universities and the research system are kept vague and invisible. In the Netherlands, universities no longer have to issue detailed annual reports since Education Minister Ritzen stripped university councils of their budget supervision authority in the late 1990s. As a consequence, overheads have become a kind of trade secret. A separate investigation by an expensive consultant would be required to make overheads visible at all.

The ridiculous transaction costs of competition, evaluation and performance management are an astounding and well-known source of financial waste, but nonetheless the system remains unaltered. For the average academic, the promise of efficiency is a bitter joke.

### The Adoration of Excellence: Everybody at the Top!

Dutch research policy is obsessed with top researchers, top departments and excellent universities. Only the best is good enough and only for the very best can we spare a dime (and then solely at the expense of the others). The ‘top’ comprises those with a plethora of publications, the clever networkers who know how to put themselves in the spotlight and how to organise large-scale research funding. Meanwhile the really big chunks of research funding are not shared in the hard-as-nails open competition at the bottom, but rather in the ad hoc programme funding for which you need to belong to the right networks. Subsequently, acquired large projects provide a hefty advantage when it comes to acquiring even more subsidies. In this way, the managerial university strengthens the dubious Matthew Effect in science (Merton 1973) even more, but now in a financial way: he who has (money), will receive even more (money) (Landsman 2013).

In the culture of winners, no-one cares what is consumed to keep this fire going. Star players often outsource their teaching duties to underpaid temporary workers. The time thus saved, can then be utilised for the expansion of their own research empire. They demand more money or better research facilities, even to the detriment of their more docile colleagues. Thus, in times of great scarcity for universities, ever increasing sums go to well-‘hyped’ stars, whilst doctoral students, postdocs and other employees with bad contracts and dismal academic career prospects perform the real toil. The managerial university itself cannot judge the intangible ‘excellence’, but believes blindly in its existence and in the assessment system’s ability to identify it, and is terrified it might miss some ‘excellent’ opportunity. Meanwhile, being one of the ‘top’ is mainly a matter of successfully managing a self-fulfilling prophecy and disguising the costs.

### Contentless Process Management

In essence, for management a university is like a company (or any other type of organisation). The required number of annual credits and doctoral dissertations are pre-set in budgets as *production targets*. ‘Management is a profession’ and ‘university management needs to professionalise’. With ‘professionalise’ they actually mean that academic professionals must *de*professionalise: they have to be demoted to executors, subject to a strict regime of supervision by *another* group of professionals: educational experts, marketing and communications staff, lawyers, real estate managers, auditors and, at the top, the professional university administrators. What is important is ‘the process’, not the objectives. The objectives are, after all, obvious: output, quality, efficiency, excellence.

The Wolf arrived in sheep’s clothing: management claims to be here to help the poor academic in these tough times of budgetary constraints. The poor academics will be relieved of the burden of administrative jobs, of the mass of paperwork, of endless meetings, and can hence concentrate on their real task. But whoever lets the Wolf in has to work each weekend through the piles of paper generated by organised mistrust and ends up writing reports and petitions to convey that this sheep is not yet ready to be slaughtered. A pet example are the extensive teaching documentation systems, in which lecturers have to record all kinds of administrative details about courses, including how each exam question relates to specific learning goals. Such petty control systems have nothing to do with ‘quality’ and only foster cynicism.

VU University Amsterdam – dubbed the ‘biscuit factory’ by academic activists – is a case in point for how this all went awry. This university has become increasingly viewed as a professionally managed company producing publications and degrees of tolerable quality at the lowest possible cost.^9^ Knowledge that is not compatible with this ‘factory logic’ is in for hard times. Consequently, management is not a neutral intervention, but has major consequences for the nature of universities’ activities. Its ‘vision’ leads to ‘big is beautiful’ policies, separation of teaching and research, a preference for instrumental skills aimed at specific labour markets and for profitable research which latches on to the next hype and does not approach its funders too critically.

Administrators of provincial universities aspire to be little Oxford, little Harvard or little Cambridge, but have no notion of the problems these places face: the rancour, the poor employment terms for those at the bottom of the organisation, the workload, the concentration of power. The social consequences, such as the formation of elite cliques, nepotism and the extreme inequality in this type of academic system, do not interest them at all. This does not prevent universities from introducing ‘top degree programmes’ in colleges based on the American model, or top research institutes with large industrial interests. At the lean Dutch universities there are no funds for these types of projects, which means that budgets have to be created; in other words, they have to be requisitioned from the rest of the organisation. This leads to prestige projects of which the manager can boast. That the permanent lecturers at these university colleges have no (or hardly any) time for research is not mentioned. And so the Netherlands now also has *wannabe universities*: megalomania with a small middle-class budget and a wake of destruction (cf. Tuchman 2009).

In the meantime, the managerial university’s system ensures that the administrators continue to believe in the occupation through extensive confirmation and lavish rewards. They receive a top salary and a car with a private chauffeur (‘If they treat me like this, I must be very special’), grow detached from the workplace and end up in a highfaluting administrative level – from the Dean through the Executive Board, the Supervisory Board, the Interuniversity Association up to the Ministry of Education, and all their surrounding moonlighting visionaries – where they all constantly parrot each other. Blatant failure, such as botched mergers between non-university higher education and universities, is no reason for critical reflection or even a delayed career. In the middle of fiasco, the managers are already busy with the next round of megalomaniac plans. If the academic staff becomes too obstreperous to carry out its policies, management flies in a contingent of well-armed crisis managers from another business sector. Critics of the policy are invited ‘for a talk’ about their irresponsible conduct and are blamed for causing reputational damage. Whoever is not with us, is against us. An important part of ‘managing the process’ is the neutralisation of doubt. Doubt is for losers.

### The Promise of Economic Salvation

Management promises that an ‘entrepreneurial university’ (the old motto of Twente University) will provide economic salvation. By cooperating with business, it expects universities will transform their wonderful discoveries into marketable products within a few years. This promise not only exhibits a naive belief in commodified universities delivering immediate economic relief, but also a shocking reduction of social benefit to economic gain. Extreme economisation has led to a radical transformation of the academic culture (Radder 2010; Engelen, Fernandez and Hendrikse 2014). The ludicrous climax is the measurement of the ‘media value’ of newspaper articles written by academics at VU University Amsterdam. Instead of valuing a contribution to public debate, these articles are now regarded as advertisements for the own university: their ‘value’ is calculated from the advertising rates for that part of the article in which this university is mentioned.

However, universities are in no way the starting point of an innovation production line culminating in new computers and smart phones – gadgets that will deliver us from the economic crisis. What universities can do is provide part of the infrastructure that enables such innovations: highly educated people, methods, a deeper understanding of what lies behind accidental discoveries, general principles and building blocks that may one day be used by a clever entrepreneur, reflections on the socio-cultural conditions for successful societal innovations. But then again, much innovation results from the opening up of new markets, new applications, improved maintenance technologies and unforeseen combinations of social and material inventions. These are processes in which academic research plays but a minor role (Edgerton 2007). The hope of economic salvation by means of enforced innovation is a misdirected yearning for a technological fix, like taking a pill to cure an unhealthy lifestyle.

In the name of economic relief, Dutch research is now surrendered to the strongest sectors of the ‘The Netherlands Inc.’: the ‘top sectors’ of the Dutch economy. Profit-oriented enterprises are gaining control over the public research infrastructure. They are allowed to restructure research to fit a time horizon their shareholders find interesting; if needs be, to the detriment of the long-term knowledge infrastructure and of those disciplines that are not easily marketable: mathematics, minor languages, philosophy and a whole range of other essential sectors of the knowledge landscape that do not necessarily bring in money.

That which does not lead to industriousness and financial gain is outmoded and questionable. History should be replaced by company history, philosophy is at most useful for the neuroethics of office innovation, sociology only as a marketing tool. Away with culture, the fundamental questions of life and the universe, the meaning of happiness or a good life.

## How Could This Happen?

Various processes have led to this state of affairs and the majority of these we, as university staff, were party to. The colonisation of the university is a success because we cooperated *en masse* – and still do, to this day.

Divide and conquer works. The humanities blame the natural sciences that they are unashamedly absconding with funds, on the basis of far-fetched promises regarding the latest micro/nano/bio/geo/digi/geno/techno bubble. The natural and life sciences accuse the social sciences of performing worthless research with meaningless surveys. Economists scoff at philosophers about their alleged lack of earning capacity and philosophers fulminate against the supposedly unreflexive business researchers. Departments try to sink their neighbours in order to pinch their budget. If a degree programme is in danger of collapse, the academic community remains mute, while the competitors count themselves rich by taking over the students. The tenured staff member mercilessly shoves the dirty work (teaching) down onto the underpaid temporary lecturer and uses the free time to work on his fame. The ‘radical’ professor lectures on the French post-modernists, while using the citation panopticon to discipline the temporary staff. Critical philosophers publish sharp papers against ‘open office’ policies, but meekly conform to its introduction at their own institution. Today we publish a manifesto, tomorrow we pull the rug from under a colleague in the hope of gaining funds for a research assistant. Divide and conquer works because we all join in.

Our generation let the Wolf in. We wanted a university more involved with society – exit the ivory tower through the science shop – but we got a university that has reduced ‘society’ to ‘business’. We wanted accountability tools to call dozing professors and burnt-out colleagues to order. But now these tools are mainly used to tightly control junior researchers with temporary appointments.

Furthermore, we allowed these tools to gradually be stripped of all content and become detailed to a ludicrous level. At some Dutch universities, there are now classification systems for publication output with no less than twenty different types, each with a corresponding score ranging from ‘AAA journal’ publications to ‘professional’ publications. This pseudoscientific system has a systematic bias in favour of English-language articles, is based on disputable interpretations of which are the renowned journals and publishers, and it confuses a medium’s quality with that of an individual publication in that medium. All of this constitutes the umpteenth step in the process of individualisation and the erosion of solidarity. Apparently, given the growing popularity of these types of assessment systems, none of this poses a problem.

And so we voluntarily adopt management’s control system. We internalise the Wolf. We introduce these inadequate accountability tools ourselves, calculate our own h-factor, count citations, create publication lists, even if no manager requests them, because we hope to be at the top of the list when the weakest sheep are sent to the slaughter. Even before the Wolf growls, we already cringe.

Even worse, we *help* to create new, more precise, extended, superior indicators: still better and more robust citation indicators, or indicators of societal impact. We receive funding for these projects and write publications about them for journals such as *Research Evaluation*. We analyse the current management shortcomings and devise better methods. And so we concoct new indicators to feed doctors, judges, nurses, policemen and, finally, our own colleagues to the Wolf.

Academics have taken refuge behind the ultimate indefensible line of defence – the measurable economic benefit. The Netherlands as a ‘knowledge society’ − we scoff at this and know that it holds a doubtful promise, but we hope that it will work and may perhaps provide universities with some extra funds. In the meantime, we are familiar with the corruption and favouritism and with the futility of large research programmes that were supposed to realise these promises, but keep quiet because we fear that even this source of money may otherwise dry up. Tacitly, we conspire to keep silent in the naïve hope that there will be some scraps left for us and that better times will come.

We market our universities or departments with the hope that this will keep our knowledge infrastructure alive. We sack those who oppose it, or whose earning capacity is too low, make outrageous compromises about content, or compete with a commercial consultant through the covert deployment of public funds. And when it seems that this strategy will allow us to survive, the Wolf tells us that we might as well enter the consultancy forest on our own – liquidate and privatise! (A list of departments forced into commercial spin-offs could follow.) At the same time, this presents a suitable opportunity for a ‘selective round of quality improvement’ (i.e. dismissal) to the advantage of the university’s holding company, allowing the Wolf to remain in firm control.

By now, we academics no longer have any allies. Administrators accommodating a life under occupation certainly offer no support. Management keeps academic staff at a great distance. At the worst affected universities, high-level administrators have very little contact with the work floor and have learned to ignore staff complaints. Supervisory Boards recruit from the same administrative level and are even more remote from the problems at the bottom. In the increasingly hierarchical university, an average academic staff member has little or no say, after academic self-governance was all but abolished at most Dutch universities in the 1990s.

There is no societal support either. There is no public support to match university funding to its performance, except for hobbyhorses or casual hypes. Politicians say that science and education are important, but eventually find other matters far more pressing. They cannot sell research and higher education to the electorate and are unable to package them into sound bites that are well received in the spectacle that is TV. Why would citizens support scientific research and scholarly inquiry anyway? “It is only there to bring in money, isn’t it? Let industry pay for it then – it will benefit them at a later stage anyway. Or, let the highly educated pay for their education themselves. An education is, after all, an individual investment, which later pays itself back royally”.

And the citizen is right: the Dutch university is no longer there for the whole of society. No more science shops, no public university, no university as a platform for uplifting the people, but instead privatised knowledge embedded in expensive patents, published in unaffordable and exclusively English-language academic journals aimed at international colleagues and businesses. Dutch-language academic journals on affairs relevant to this society have been abolished, sacrificed on the altar of the international academic league tables. We organise top English-language degree programmes aimed at the international market of better students, with fatter grants, generating more income for the university. The entrepreneurial university is now primarily interested in lucrative ventures, not citizens’ concerns. The gambit of the Netherlands as a knowledge society has failed and has now turned against us.

We cannot count on the support of students either – stuck as they are between loans, performance grants and meagre labour market perspectives, or mesmerised by the lure of a consumerist life. Why would they have sympathy for the demanding academic who delays their ascent to a diploma, who places intellectual obstacles on the zigzag way to vital credits? When management tackled students and turned them into well-drilled, debt-laden, labour market-oriented contract-performance machines, we did not lift a finger. They too are right.

So, there we are: cowards, beggars, petty thieves and collaborators. Together, we face the Wolf alone.

## The Alternative: The Public University

Even if the Wolf denies it, alternatives to the current managerial university do exist and articulating them is not even that hard. One alternative we reject outright: we do not want to return to the mythical ivory tower. Pleas for a return to bygone golden times and the beauty of academic autonomy are misdirected.^10^ Those times were not as blissful as some nostalgic dreamers suggest: the unequal access, the nepotism, the endless meetings, the ineffectuality, the fiddling, the mustiness … The ivory tower has become a childish daydream, a mirage reminding us of the curious tales of utopian socialists. Not only is it unclear how this ideal academic society should exactly function, but also how we should attain this state of bliss. Indeed, these kinds of pleas do our case more harm than good.

We much prefer the project of a public university aimed at the common good − and at the careful deliberation of what comprises ‘the common good’.^11^ This does not imply a reduction to ‘applied science’. Fundamental research (not to be confused with autonomous science – science has never been ‘autonomous’) is of eminent social concern. The outcomes of this research form a vital resource we can draw upon if the future turns out to be totally different than our short-term extrapolations had suggested. Currently, historical and socio-cultural Islamic studies are seen to be topical and important, but thirty years ago the Wolf would have regarded them as economically irresponsible hobbyhorses.

As universities we are looking for novel ways of social cooperation, in which we offer (world) citizens and their organisations our knowledge, even if they cannot afford it. We are not the start of a production line of a profitable gadget factory, but a knowledge commons: a shared, organically growing garden of know-how and wisdom from which everybody may learn according to their needs, and to which everybody may contribute. This knowledge commons teems with recipes, systems, interpretations, collections, methods, criticisms, arguments, data files, images, utopias and dystopias, experiences, measurements, and countless other results of scientific work that are all beyond the horizon of the gadget factory. This archive is chaotic but hyper-fertile, a proliferating garden bursting with problem-locating and problem-solving capabilities accrued throughout the ages (and not only over the past five years, as the Wolf’s librarians sometimes seem to think). The archive tells us who we are, who we *could* be and how we could get there. Our task is to share the produce of this garden and to cultivate it with as many people as possible, not to cultivate standardised minds at the lowest possible cost.

We are looking for new allies, new students and new partners in this public space. Not because they have money, but because they have something to say. We will only request public support after we have once again proven our public interest (which the Wolf systematically confuses with *economic* interest). In the meantime, we revitalise our research by showing the Wolf’s suspicious controllers the door and replacing the publication rat-race with more meaningful, slower-paced and more considerate research (Pels 2003). Perhaps we will then find the time to actually read what our colleagues have written. We will also simply allow the expensive and arrogant top-talents to follow up on their threats and depart to more promising foreign shores. We will replace them with a multitude of researchers with normal salaries, who prioritise intellectual content and public interest over reputation. We will tell students that they study to become good scholars and responsible members of society, not for the sake of a highly-paid job at the end of the diploma ceremony. We will once again tell them that they are primarily learners and citizens, not consumers.

Contrary to what the Wolf claims, it is not really that difficult to formulate concrete proposals to realise this model and avoid sliding back into the Wolf’s den. We present twenty provocative first moves.

### From Hierarchy to Having a Voice

The *conditio sine qua non* for all university renewal is to breach the current hierarchical top-down structure and move towards a form of administration in which academics, students and supporting staff can have a voice and can decide on the matters they know best. In the Netherlands, this would formally require a change in the law on higher education. However, nothing prevents departments and faculties from already starting to experiment with new, informal forms of participation and representation. They could, for instance, follow those US universities where faculty has retained a real voice in the appointment of new staff.

### Administration as Support Staff

The campus organisation becomes a general and technical support service comprising the administrator, the janitor and the cleaner, all paid by the same organisation and not by some semi-legal precarious employment agency. This general and technical support service becomes an organisation stimulating the development and sharing of knowledge, instead of a knowledge plantation with supervisors maximising ‘valorisation’ through Taylorism and control.

### Limiting Wasteful Control Systems

A substantial simplification and reduction of control systems will free up resources for academic work. Not more than 10% of the time is to be spent on administrative overheads, such as audit procedures, filling out teaching documentation forms, reports for external reviews and research proposals. What cannot be done in this 10% should not be done.

### A Ban on Mergers

The current scale of Dutch universities is large enough: a ban on mergers within and between universities saves staff and money, and preserves motivation. Stimulation of cooperation, instead of managerial scaling-up. Larger institutions lead to a reinforcement of management.

### An End to the Own-University-First Policy

Inter-university cooperation is undertaken by means of the academic staff’s cooperation and not via scheming top managers who sabotage fertile national academic cooperation because they themselves cannot use it to boast, or because it does not fit within their megalomaniacal conceptions.

### No Wasteful Competition Between Universities

Cooperation, as exemplified in the Dutch national research schools, becomes the norm. Students are mutually referred to courses at other universities (instead of being hijacked), with the interest of the students at heart and not that of the institutions. Top managers’ overfunded prestigious hobbyhorses are immediately discontinued and the funds redirected for the benefit of the primary processes of teaching and research.

### A Ban on University Marketing

The discontinuation of all PR and marketing departments. No more wasting of money on glossy ads, no expensive, prestigious information campaigns with matching branded presents, no more expensive upgrading of the corporate identity, closing of the university branding shop in the city centre’s top location. Henceforth, these types of projects are considered wasteful and a misuse of public means intended for education and research.

### University News Media for Public Debate and not for PR

Strengthening of the independent university news media in which the entire academic community can discuss university matters without fear of repercussions. Discontinuation of the management’s corporate glossies and websites that pose as news media.

### Return of Detailed Annual Reports

The university administration is accountable to the academic community and not vice versa. The overheads on all managerial levels are therefore disclosed in detail. Financial control reverts to the academic community, supported by appropriate financial reporting.

### No Real Estate Speculation and Discontinuation of Holding Companies

University buildings become collective property on which there is no speculation. Buildings will no longer be used for money-earning purposes by renting them out for commercial activities, but should be reserved for academic and public use. Universities are public institutions and not enterprises. University holding companies have deservedly acquired a bad name due to legally questionable employment agencies, real estate speculation and spurious consultancy businesses.

### All Academic Staff Members Teach

No exemption for arrogant top talents: *all* academic staff members devote at least 20% of their time to education. Local education should also benefit from the research of the frequently publishing, international congress jet set. Nobody only teaches – no more passing down to the poorly-paid and precariously employed teaching proletariat − and everybody has the right to research time. The same hard, basic norm for *all* groups, including managerial academics and members of (education) committees, across disciplines.

### Free Education

Learning and studying are free and lifelong learning is made possible, including following multiple degree programmes. We immediately abolish the expensive administrative units policing study-progress. This does not mean that studying should be subsidised indefinitely, but we should allow talented students to develop themselves in more than one discipline. In the university’s knowledge commons, learning is a collective right, even if you are older, or cannot afford to take the financial risk of a study loan. The more the fruits of our academic garden are shared, the greater the problem-solving capability of the society, the greater the country’s wealth.

### An Upper Limit to the Student Population

A maximum student population for a specific degree programme, proportional to the size of the available lecture rooms and in relation to the available lecturers for the courses. If the population increases, the time has come for an additional programme or for referral to another institution. When universities become too overcrowded, it is time for a new university and not for further false economies of scale.

### Separation of Purely Vocational Training

Renewed separation between narrow, instrumental, career-oriented higher education at vocational schools and higher education for expert academics and broadly oriented public professionals at universities.

### The End of ‘Productivity’ as a Research Assessment Criterion

To prevent the production of senseless publications and the abuse of the publication system, the criterion ‘productivity’ (the number of publications per full time staff) is erased from all research assessments. You publish when you have something new to say and not because you hope to get a promotion or research funding. What counts is the quality of the content and its contribution to scientific and social debates.

### Introducing the Sabbatical Year

Each academic (also those who first work temporarily at one university for four years and then temporarily for two years at another) has the right to a sabbatical year once every seven years. This year is spent on expanding and renewing knowledge and insight for education and research purposes.

### Discontinuation of Matching

Those who want to finance research, finance it completely and do not misuse their position of power to obtain greater control over the university than what they have paid for. Matching drains resources from vulnerable parts of the knowledge infrastructure through inappropriate diversion of public means.

### Separation of Content-Oriented Assessment and Remuneration

At a public university (and according to all codes of ethical conduct) *direct* relations between the content-oriented assessment of teaching and research, on the one hand, and financial interests, on the other hand, are unacceptable. Output financing on the basis of acquired course credits, the bonus for the approval of dissertations, and the patenting of publicly financed research results are immediately discontinued. The strengthening of market-oriented research occurs at specific *market-oriented* research organisations, not at public universities.

### Social Participation Instead of Commercial Control

Social organisations and citizens, in conjunction with the university staff, are invited (and receive a modest compensation) to help articulate the university’s public duties.

### Support of the Knowledge Commons

Strengthening the public accessibility to knowledge: reintroduction of science shops, open access publishing where possible and meaningful, public lectures, freely accessible digital learning environments, publicly available workplaces (fab-labs).

Clearly, an alternative academic future does exist, and concrete options can be articulated. Yes, the unread publication output, the universities’ advertising value in the media and the scores in meaningless rankings will decrease. We believe this would be a good indication of a successful renewal. Proponents of the status quo will possibly find our proposals absurd. This is fine – we find the status quo absurd.

## If Arguments no Longer Count, it is Time for Action!

The managerial university is impervious to arguments. Only what the buddies in the managerial cloud think is important. There is a plethora of critical essays, pamphlets and manifestos, but management simply shrugs these off as irrelevant, as the final convulsions of those dreamers still living in yesteryear, or perhaps as the lamentations of losers, understandable, but not to be taken seriously. For the time being, they find listening harder than ignoring and thus they continue to boast about their next prestige project.

These poor manifesto-writing academics truly believe that they can change the world with the mighty pen. We carefully analyse the attributes of the New Public Management (De Boer, Enders and Schimank 2007) and quarrel over the exact meaning of the term (Hood and Peters 2004). Should we call the escalating control Taylorism, McDonaldization, supermarket university, or is it rather proletarianisation (Hayes and Wynyard 2002)? Or is ‘neoliberalism’ perhaps more fitting? For the past quarter century, we have been documenting this process of decline and have been quarrelling about what the Wolf should be called. In the meantime, we are steadily slipping away.

No, it is high time to offer resistance. Only through broadly-supported, collective resistance will we be heard. Only by shaking off our fear can we reflect on our universities’ future, collectively and on an equal footing.

What can we do? Let us try to learn from the many who lived (or are still living) under an occupation and discover which fruitful and less fruitful strategies they developed. What are our options?

### Exit

An individual academic can, like generations of poor souls before, emigrate to the New World in the hope of better prospects. Many are making this choice (see the Dutch brain drain of young academics who no longer want to work at a university). This time around, the New World is not such a good idea, although it seems that Scandinavia is not yet completely occupied.

One can also try to go one’s own way by becoming a publicist or a consultant. This survival strategy may be viable for a select few, but it hardly generates new science or scholarship. Or one can go elsewhere and then try to return with the capital that is still valued in the current university, such as experience in The Hague, Brussels or the business world (as this is perceived as fundraising potential) or a shiny medal from a New-World top club (because this looks so good on the website). Although such an exit ensures a tail wind for returning rising stars, it does not, of course, solve the problems of those who stay behind.

### Legal Action

One can also resort to the courts. There are still possibilities for legal action, specifically by means of labour law, if the management goes too far. In a few cases, personnel at Dutch universities have booked some success by threatening to go to court, but in general, legal action is not really effective. An abused temporary employee will receive no more than an individual severance fee, and a reputation as a troublemaker. Even the Dutch trade unions have not succeeded in preventing the most serious forms of misuse (self-employed lecturers, university employment agencies, intermittent unemployment to avoid having to provide permanent employment, late payment for already presented lectures to avoid the obligation of having to provide a fixed contract, etc.).

### Muddling Through and Work-to-Rule

We muddle through while trying to survive the occupation, even if we have to lie and deceive. We urge our colleagues to refer more to each other’s work to make the indicators look better. We carefully orchestrate our reports for external reviews, hiding all possible weaknesses during an inspection (if necessary, in cooperation with the examining inspector, a professorial acquaintance from abroad). We also create fake products, repetitions of publications to embellish the score, worthless papers buffed-up to resemble important contributions to whatever. Or we supply nonsensical answers to ridiculous questions in senseless control systems. On the instruction of their bookkeepers, scientists lie *en masse* when writing time on forms which presume that nobody works more than eight hours a day and never on Sunday. This minor resistance does not really produce structural change. On the contrary, this muddling through lubricates the occupation machine, which would otherwise collapse under its own absurdity.

Working to rule is one way of exposing bureaucratic absurdity. Researchers who have to fill in their hours (especially everyone who works on EU-funded projects) could refuse to continue to lie about how much and when they work. In general, systematic working-to-rule actions may be of limited relevance to academic work.

### Sabotage

Weavers going to work in industrialised production offered resistance by throwing a clog (sabot) into the looms. Those sheep caught committing sabotage are immediately sent to the slaughter by the Wolf – this is a very risky strategy. But perhaps the time has come to replace our collaborating muddling with a more conscious undermining of ridiculous control systems, for example, by filling them with useless nonsense. A case in point is the obligatory entering of learning objectives and marking matrices in teaching documentation forms. One still finds established professors at universities claiming that they refuse to cooperate with these kinds of systems, but the semi-permanent lecturer cannot afford this. Yet, some procrastination, lost data or intricate complications may be very suitable instruments for sending the occupier off none the wiser and, in any case, will lead to less docile collaboration (Torfs 2014). Sabotage remains dangerous, however, and there is no guarantee that the individual saboteur will not go too far and discredit the whole cause.

### The Collective Refusal

Refusing to cooperate with senseless control can only be done collectively, after deliberation and with support for those who are caught out. Quite a few ridiculous systems could collapse through a simple collective refusal, such as the inadequate registration systems of annual research results, the PR instructions to boost your citation score, the course documentation systems, and the international university rankings (e.g. German universities’ collective refusal to continue to supply information for rankings). This type of small-scale resistance requires some organisation and solidarity. Is this still feasible at the current universities?

### Trade Union Actions

After university democracy was replaced by an employees council at most Dutch universities, only the trade unions remained as the collective representation of the ‘staff’. However, in the employees council, the trade unions only had the right to discuss labour conditions and no longer the organisation of academic labour itself. Since primarily the older generation still tend to be trade union members, the classic ‘service-oriented’ trade unions focus predominantly on their interests. The large university trade unions have for decades concentrated on the preservation of (permanent) employment, pensions and procedures for voluntary severance, instead of on the exploitation of young academics, the occupation, and the managerial university.

We could have predicted this error, because the labour movement had previously made the same mistake. This is called ‘the trap of co-management’, otherwise known as repressive tolerance: the promise of participation, which is limited to the marginal phenomena of a process about which you have no fundamental say, while management can maintain that you were allowed to participate in the discussions. Nonetheless, labour movement history also demonstrates that no structural progress is possible without collective organisation. Either our present unions wake up (and we awake in our unions), or we have to establish new ones.

What do trade unions do? Trade unions mobilise *solidarity*. Remember what that was? Solidarity means mutual support, also if you yourself came off scot-free. It means that people at universities who have not yet been hit as hard by the occupation (e.g., at Radboud University Nijmegen) also take action when other universities sink into the abyss (e.g., VU University Amsterdam and the University of Amsterdam), instead of passively standing by and watching, which is what they do now. Solidarity of employees and their trade unions ensures that, through collective action, resistance once again becomes a right and that management exploitation (such as kamikaze contracts, permanently temporary work) becomes illegal again.

### Mass Demonstration

Large demonstrations are ‘out’ in the Netherlands, but are back at other European universities (the UK, France, Austria). Demonstrations hack away at the legitimacy of power by highlighting collective refusal, but then only if they are on a grand scale (Tahrir), or prolonged (Plaza de Mayo); if not, they are counterproductive. They combine well with mediagenic, delegitimising action: the frivolous act of laughing at the naked emperor. No respect for collaborating administrators under the Wolf’s occupation, just mockery and contempt. An increase in the Shanghai ranking is no reason to serve cake to the Wolf but rather to throw it at them!

### Contra-Indictors as Counter-Measures

The panopticon of control and accountability reports much but omits far more, intentionally or unintentionally. We ought to expose the excesses of the occupation better, respond with *alternative* rankings and *contra*-indicators, *counter-*measures. These measures aim at systematically making visible and comparable quite other things than our ‘output’: they concern waste, overheads and destruction of resources. For instance:

- A (Dutch) ranking of university overheads. Often, in the case of external contract-funded projects, researchers have to pay a percentage of overhead to the universities and faculties where they will be based. This percentage varies among different institutions. Its size can be revealed by simply asking one’s colleagues. Rotterdam reports 50%! Is there a lower bid? Then we allocate the contract to our friends in that institution.
- An overhead ranking of financing instruments: NWO (the Dutch Organisation for Scientific Research) can do it for 25% overhead (Van Arensbergen, Hessels and Van der Meulen 2013), but universities can do it for less (Van der Burg 2012). A ranking of the largest PR departments, the largest external review overheads, the highest manager remuneration, the most expensive boardroom, the most costly branded products.
- Filling in hours and claiming payment for overheads (which is, after all, usual in commercial companies): systematically revealing the full costs of, for example, assessment of research proposals, external reviews (participation and preparation), completing audit reports, teaching records, updating of the teaching portfolio, evaluation committees … Always revealing the cost, or even sending an account. ‘Replying to your administrative control has cost me two working hours. This is equivalent to so many Euros based on the contract-funded rate. Plus 50% overheads, naturally’.
- Revealing university employees’ income inequality (including the temporary employees), now and in the past. Including the bonuses, of course.
- Drawing up a ranking of the most expensive failed prestige projects and of the most costly complete and semi-mergers, beginning with those between universities and non-university higher education institutions.

### The Strike

This is the ultimate weapon, very unpopular in a country like the Netherlands where deliberation is the norm. The problem with strikes in the public sector is that you gain almost no sympathy. In this case, we would seriously disadvantage the students, already weighed down by performance grants and/or student loans. In addition, many academics already fear missing out on citations if they were once not to work on a weekend. Striking is also contrary to the intrinsic motivation of many colleagues – the still strongly present feeling that, despite everything, an academic profession is also a vocation.

Here too we can learn from the labour movement. The core of a strike is refusal, the discontinuation of tasks, which can take many forms, such as work-to-rule strikes, payment strikes (not collecting payments for societal services rendered), administrative strikes, etc. Students have major leverage here: a collective pay strike through a refusal to pay tuition fees.

### The Contra-Occupation

The occupation of university buildings is a classic academic form of action, generally carried out by students who do not have to care for a family in the evening. It is also a risky form of action: an occupation is illegal and can lead to arrests, and even to a violent eviction by the authorities. In addition, there is a considerable risk that the occupation of a building becomes a goal in itself, whereby the occupiers become isolated from their rank and file supporters. Nevertheless, it is a mediagenic, highly-charged symbolic action: the university is ostensibly reclaimed from the Wolf.

### Parliamentary and Political Action

Dutch universities only really attract political interest when student loans and grants are at stake. Science policy is normally too technical, too difficult to wrap up in simple sound bites, or in the clichés of the daily news soap opera. Politicians will only act if we ourselves create newsworthy facts: report the false promises, misuse of resources, corruption, conflict of interest, arrogant leaders, Kafkaesque bureaucracy, scandals. But we must also show that these are not isolated incidents, but the consequences of structural flaws in the hierarchical managerial university. We need to think along with politicians about a new form of governance, which is required for the realisation of the public university.

Thus far, eleven examples of actions with which emancipatory movements have attempted to shake off their occupier. It should be clear that this may be an arduous, never-ending journey. At the same time, it is evident that the need for collective resistance is high, because management could not care less about arguments and manifestos. Only one conclusion is possible:

Workers of all universities: unite!^12^

## Footnotes

1 This article is a slightly updated and edited translation of the Dutch original (entitled ‘Het academisch manifest: van een bezette naar een publieke universiteit’), which appeared in *Krisis: Tijdschrift voor actuele filosofie* 2013 (3), pp. 2–18. We are grateful for helpful commentary on that version by the *Krisis* editorial team, in particular René Gabriëls. We would also like to thank Jan and Ilse Evertse for translating the Dutch text into English, and Mark Brown and Peter Weingart for comments on the English version.

2 According to accepted clinical norms, a quarter of Dutch professors of medical science (especially the younger ones) suffer from burn-out (Tijdink, Vergouwen and Smulders 2012).

3 See, e.g., Ritzer (1998); Graham (2002); Hayes and Wynyard (2002); Bok (2003); Washburn (2003); Evans (2005); Schimank (2005); Boomkens (2008); Gill (2009); Tuchman (2009); Radder (2010); Krijnen, Lorenz and Umlauf (2011); Collini (2012); Sanders and Van der Zweerde (2012); Dijstelbloem et al. (2013); Bal, Grassiani and Kirk (2014); Verbrugge and Van Baardewijk (2014).

4 See Lorenz (2006 and 2012); Krücken (2014). In line with the situation in most European countries, we assume that universities are, or should be, characterized by a unity of teaching and research.

5 We are aware that these causes are related to wide-ranging political and socio-cultural developments, such as the rise of a neoliberal politics and worldview, and occasionally we refer to these broader developments. By focusing on what is happening in universities, we think that we can best identify and further the required remedies.

6 However, if those ‘stupid’ books by Foucault, Bourdieu and Derrida are, in fact, the most cited in their discipline (Heilbron 2011: 17–19), *this* has no influence on the prevailing policy.

7 Some recent changes in research evaluation policies suggest that productivity will become less important (see Ministerie van Onderwijs, Cultuur en Wetenschap 2014), but we have to wait to see how this will work out in practice.

8 The high appreciation scores by the students at VU University Amsterdam plummeted when the number of students increased spectacularly (Funnekotter and Logtenberg 2013). Similarly, when the programme in communication science at the University of Amsterdam ‘successfully’ attracted hundreds of students in the 1990s, the quality dropped so much that this led to a press scandal and students threatening legal action. Currently, the winners of the competition present lectures in parallel rooms, projecting the lectures on a second screen, because the course is so over-subscribed that the students no longer fit into the lecture halls. It is feasible, but it is *not* an improvement of quality.

9 The drama at VU University Amsterdam received extensive media attention (Radder 2012; Verontruste VU’ers 2012; Funnekotter and Logtenberg 2013; Ten Hooven 2013).

10 Jeff Lustig (2005: 24) even argues that, in the 19th and the first half of the 20th century, American universities were understood as dedicated to the public good rather than seen as an ivory tower.

11 Note that ‘a public university aimed at the common good’ does not necessarily coincide with ‘a publicly funded university’.

12 As we are preparing this manuscript for publication (early March 2015), some hopeful signs of change are appearing. Students, followed by faculty, occupied two buildings of the University of Amsterdam (see http://newuni.nl/ and http://rethinkuva.org/) and are getting support at other universities. This new movement has forcefully taken up many of the critical analyses and constructive proposals described in this Manifesto.
